# Patient Selection in Deep Brain Stimulation: A Role for Transcranial Direct Current Stimulation to Enhance the Levodopa Challenge?

**DOI:** 10.1002/acn3.70073

**Published:** 2025-05-19

**Authors:** Lukas L. Goede, Patricia Zvarova, Bahne H. Bahners, Andreas Horn

**Affiliations:** ^1^ Center for Brain Circuit Therapeutics, Departments of Neurology, Psychiatry, and Radiology Brigham and Women's Hospital, Harvard Medical School Boston Massachusetts USA; ^2^ Department of Neurology With Experimental Neurology Charité—Universitätsmedizin Berlin, Corporate Member of Freie Universität Berlin and Humboldt Universität Zu Berlin Berlin Germany; ^3^ Einstein Center for Neurosciences Berlin Charité—Universitätsmedizin Berlin Berlin Germany; ^4^ MGH Neurosurgery & Center for Neurotechnology and Neurorecovery (CNTR) MGH Neurology Massachusetts General Hospital, Harvard Medical School Boston Massachusetts USA; ^5^ Institute for Network Stimulation, Department of Stereotactic and Functional Neurosurgery University Hospital Cologne Cologne Germany

**Keywords:** deep brain stimulation, movement disorders, parkinson's disease, transcranial direct current stimulation

## Abstract

Dopaminergic medication and deep brain stimulation (DBS) improve motor symptoms in Parkinson's disease (PD), but levodopa response alone may not predict DBS outcomes. We retrospectively analyzed 19 PD patients undergoing levodopa challenges with and without prior transcranial direct current stimulation targeting a defined *PD response network*. Levodopa improved motor performance more after tDCS than sham (41.72% vs. 31.52%; *p* < 0.001). In ten patients who later received DBS, the combined levodopa‐tDCS response accounted for DBS outcomes (*p* = 0.02). These findings suggest that targeted tDCS enhances levodopa effects and may be of potential use to optimize DBS candidate selection.

## 1 Introduction

Since the 1960s, levodopa has been the cornerstone of symptomatic treatment of Parkinson's disease (PD), enhancing synaptic dopamine transmission [1], and leading to motor symptom improvements [2]. For patients with advanced PD and significant motor complications, deep brain stimulation (DBS) of the subthalamic nucleus (STN) or globus pallidus internus (GPi) has emerged as an effective therapeutic option, often allowing a reduction in levodopa dosage and providing substantial clinical improvement [3]. Despite its benefits, not all patients experience the same degree of improvement with DBS, making effective patient selection crucial. Although the preoperative levodopa challenge is commonly used for DBS patient selection, recent multicenter data [4] and theoretical work [5] have questioned whether the levodopa response alone is a sensible marker for DBS success.

Previous studies have identified [6] and confirmed [7, 8] a specific brain network, termed the PD response network, which, when stimulated, is associated with maximal motor improvements following DBS in PD. A recent double‐blind, sham‐controlled study was able to demonstrate that this same network may be stimulated non‐invasively, using transcranial direct current stimulation with a multi‐electrode approach that was tailored to maximally impact this specific network [9]. Notably, results of this study also suggested that the effects of tDCS correlated with DBS outcomes in a subgroup of patients that underwent DBS surgery, suggesting that tDCS may bear potential to serve as a tool for estimating DBS efficacy before surgery.

In the present study, we revisit these findings and investigate the interplay between acute responses to levodopa, tDCS, and DBS. Specifically, first, we hypothesize that the applied tDCS approach might have a priming effect on the network that could lead to enhanced motor improvements following a levodopa challenge. Second, we explore whether the response to tDCS and the response in the levodopa challenge might explain additional amounts of variance in DBS response, i.e., could serve as independent predictors for patient selection.

## 2 Methods

Clinical data were retrospectively analyzed from 19 Parkinson's disease (PD) patients (mean age: 60.1 ± 6.1 years; mean Hoehn & Yahr stage: 2.4 ± 0.7) who previously participated in a pre‐registered, prospective, double‐blind, cross‐over trial investigating the effects of network‐targeted transcranial direct current stimulation (tDCS) on motor function [9]. In the original study, published separately, each subject underwent two sessions (active tDCS and sham stimulation; electrode montage shown in Figure S1) administered in a randomized order with a minimum inter‐session interval of 24 h. For the levodopa challenge, patients received 200 mg of immediate‐release levodopa. Notably, this assessment took place 60 min after tDCS (or sham) and was not part of the pre‐registered endpoints of the initial tDCS trial. Motor performance was assessed using the motor part of the Movement Disorder Society‐Unified Parkinson's Disease Rating Scale (MDS‐UPDRS‐III), with levodopa‐induced improvement quantified as the reduction in scores from baseline to best “on state” post‐administration. Importantly, clinical raters were blinded to whether patients received active or sham stimulation prior to the levodopa challenge. Additionally, follow‐up clinical records were obtained for 10 patients who subsequently underwent subthalamic nucleus (STN) deep brain stimulation (DBS) surgery at Charité—Universitätsmedizin Berlin. DBS improvement scores were assessed in the state without dopaminergic medication (DBS ON, MED OFF) and compared to the MED OFF assessments that were carried out on days of active tDCS sessions (pre‐tDCS stimulation OFF).

Clinical improvement scores were compared using paired two‐sided t‐tests to assess differences in levodopa‐induced motor improvements. Pearson's correlation tests were performed to evaluate the association between MDS‐UPDRS‐III improvements after DBS and those following active tDCS and levodopa, respectively. A linear model was fit to assess how much variance in DBS outcomes could be explained by levodopa‐induced and tDCS‐induced motor improvements . Statistical analyses were carried out in MATLAB (version R2023b). The study protocol was approved by the Institutional Review Board at Charité‐Universitätsmedizin Berlin, and all procedures were performed in accordance with the ethical guidelines of the Declaration of Helsinki.

## 3 Results

**FIGURE 1:**
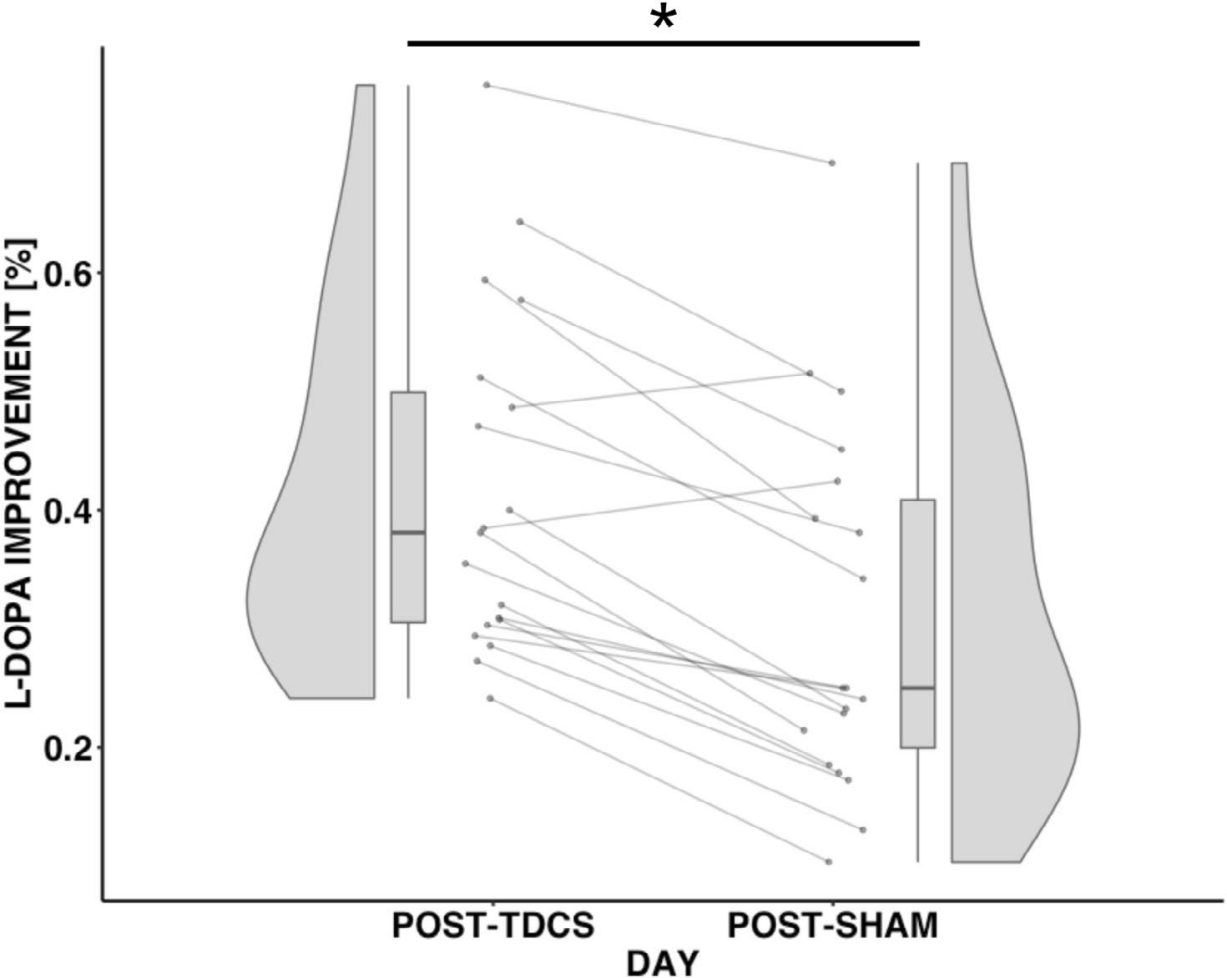
Violin and box plots comparing the percentage improvement in MDS‐UPDRS‐III scores after intake of 200 mg levodopa acquired on two separate days following transcranial direct current stimulation (tDCS, left) and sham stimulation (right). The order of tDCS and sham stimulation sessions was randomized, with a minimum interval of 24 h between sessions. *p < 0.001 in a two‐sided paired *t*‐test.

Before the levodopa challenge, the 19 PD patients had similar baseline MDS‐UPDRS‐III scores on both tDCS (34.95 ± 12.38) and sham days (35.53 ± 11.32; *t* (18) = 1.07, *p* = 0.29). Following 200 mg oral levodopa, MDS‐UPDRS‐III scores significantly decreased on both days. On the active tDCS day, scores reduced to 19.74 ± 7.04 points (41.72% improvement), and on the sham day to 23.68 ± 6.78 points (31.52% improvement). The improvement was significantly greater following active tDCS compared to sham stimulation (*t* (18) = 7.11, *p* < 0.001) (Figure 1).

**FIGURE 2:**
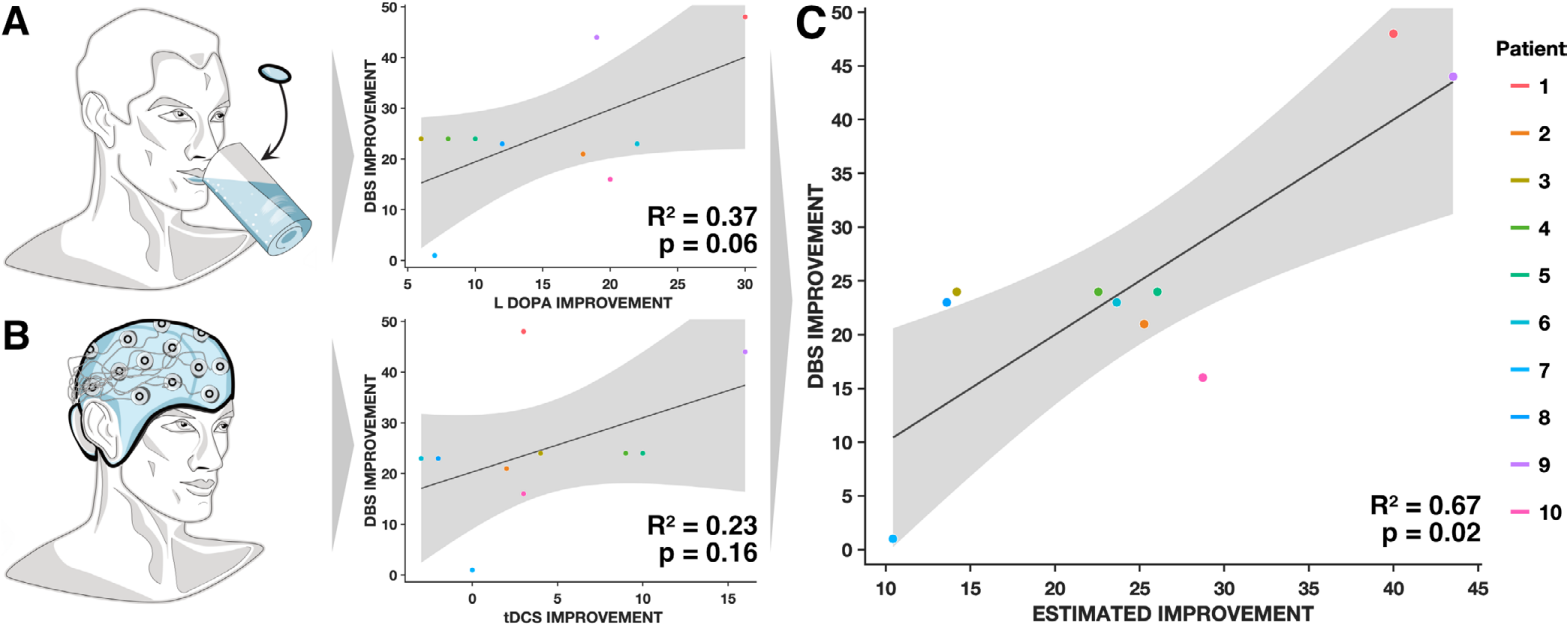
Clinical Improvements following levodopa intake and tDCS associate with DBS outcomes. (A) When analyzed alone, levodopa improvement positively, but not significantly correlated with DBS improvements. (B) Similarly, when analyzed alone, tDCS positively, but not significantly correlated with DBS improvements. (C) When analyzed in a combined linear model, the two variables accounted for significant amounts of variance in DBS outcomes. Critically, the two patients that had very strong improvements following DBS were each singled out by one of the two regressors (but not the other one) and hence correctly predicted as top‐responders when aggregating both scores.

Ten patients underwent DBS surgery outside the study protocol, on average 5.7 ± 4.47 months after participating in the tDCS trial. Their clinical improvement scores were subsequently assessed at a follow‐up visit 10.8 ± 2.94 months later. The correlation between levodopa‐induced improvement and DBS improvement was positive but only statistically significant in a one‐tailed test (*R* ^2^ = 0.37, *p*_(1)_ = 0.03; *p*_(2)_ = 0.06) (Figure 2A). Similarly, the correlation between tDCS‐induced improvement and DBS improvement was positive but not significant (*R* ^2^ = 0.23, *p*_(1)_ = 0.08; *p*_(2)_ = 0.16) (Figure 2B).

When both tDCS and levodopa improvements were included in a combined model, they accounted for significant amounts of variance in DBS outcomes (*F* (2, 7) = 7, *R* ^2^ = 0.67, *p* = 0.02). Critically, both tDCS and levodopa improvements were independent significant regressors that each explained additional amounts of variance compared to the respective other variable (Table S1).

## 4 Discussion

There are two key take home points from this post hoc analysis study. First, results suggest that tDCS using multiple electrodes in a specific array might ‘prime’ an a priori defined *Parkinson's Disease Response Network* in such a way that subsequent levodopa intake would lead to enhanced motor improvements. Second, while motor improvements following tDCS and levodopa intake each positively associated with motor improvements following DBS, these relationships were not significant for either variable alone in the small sample analyzed (*N* = 10). However, when analyzed in a joint linear model, the two variables accounted for significant amounts of variance in DBS improvements and each served as an independent predictor in the model, i.e., explained additional variance above and beyond the respective other one.

An enhanced motor improvement following levodopa intake when administered after active tDCS may indicate that this neuromodulation strategy may modulate a neural network involved in leading to treatment response by augmenting the response to dopaminergic therapy. Since the specific tDCS application [9] was montaged to maximally modulate an a priori defined PD response network [7, 10], it is reasonable to assume that components of this network may serve as a substrate in this process, which aligns with previous studies demonstrating that both brain stimulation and levodopa may facilitate brain signals coding for motor performance in PD patients [11, 12].

A recent multicenter trial raised concerns that the commonly applied ‘levodopa challenge’ is an insufficient predictor for DBS outcomes [4], and other studies could only confirm levodopa response as a predictor for short‐ but not long‐term DBS improvements [13, 14], emphasizing the need for more comprehensive predictive models. In our sample, we observed a positive, though non‐significant, correlation, suggesting that our small sample size is insufficient to meaningfully contribute to this discussion. However, while tDCS‐induced improvement alone was also not a significant predictor, when combined with levodopa responsiveness in a joint model, the two variables explained significant amounts of variance in DBS improvements. This suggests that the two variables may explain additional amounts of variance, and a combination of the two could be helpful in DBS patient selection.

We must emphasize that these conclusions are drawn from a small sample (*N* = 10) but since the sample has uniquely undergone levodopa challenge, DBS, and tDCS, we deem these results noteworthy and potentially hypothesis‐generating, nonetheless. Critically, this finding should be replicated in a larger cohort that would specifically investigate the relationship between the three treatment modalities in an independent sample. Importantly, this study did not compare clinical effects across different montage designs or varying numbers of electrodes, nor did it assess whether individual variability in the induced electric field correlates with clinical outcomes, both of which warrant systematic investigation in future research. Furthermore, simpler arrays (e.g., with electrodes along the motor strip) could likely lead to similar neuromodulatory effects.

In summary, our findings suggest a potential of tDCS using multiple electrodes in a specific array to enhance levodopa effects and improve the prediction of DBS outcomes when the two components are analyzed together. Once confirmed on a larger cohort, the approach to combine noninvasive stimulation response with levodopa response may improve DBS patient selection and, ultimately, therapeutic outcomes in PD.

## Author Contributions

L.L.G. and A.H. conceptualized the study. L.L.G., P.Z., B.H.B., and A.H. analyzed the data. L.L.G. wrote the manuscript with input and revisions from all authors.

## Conflicts of Interest

The authors declare no conflicts of interest.

## Supporting information


**Appendix S1.** Optimization of the tDCS electrode array.

## Data Availability

All data generated or analyzed during this study are included in this published article. Additional information is available from the corresponding author upon reasonable request.
