# Supplementary material for: Patient Selection in Deep Brain Stimulation: A Role for Transcranial Direct Current Stimulation to Enhance the Levodopa Challenge?
Source: Ann Clin Transl Neurol. 2025 May 19;12(8):1698–701. doi: 10.1002/acn3.70073 (PMC12343323; doi:10.1002/acn3.70073)
Supplement: Supplementary file 1 — Appendix S1. Optimization of the tDCS electrode array. [file ACN3-12-1698-s001.docx]

**Supplementary data**


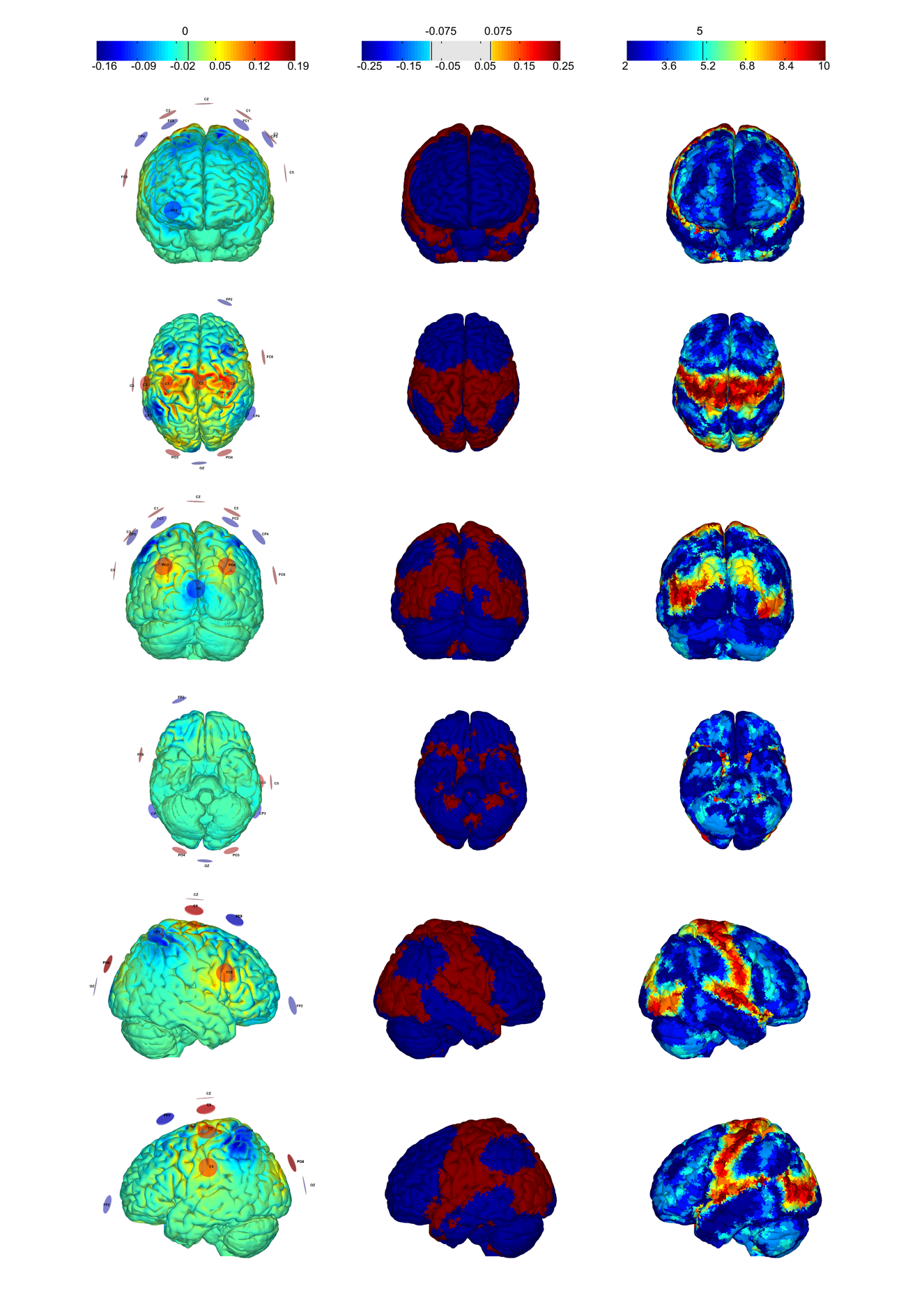


**Supplementary figure S1.** **Optimization of the tDCS electrode array.** I_Max_ = 1.044mA, 14 electrodes. From left to right: Normal component of the electric field (En, V/m), target electric field (V/m), and target weight. The multichannel montage was generated using an optimization framework based on resting-state fMRI-derived functional connectivity. The algorithm prioritized En (field component normal to the cortical surface) to maximize focality and directionality, while constraining stimulation to a maximum of 14 electrodes from 64 possible cap positions. The resulting montage achieved 97% of the theoretical optimal targeting accuracy and accounts for cortical folding and asymmetry. Displayed views highlight the modeled electric field from multiple angles.

|  | **Estimate** | **SE** | ***t*-Value** | ***p*-Value** |
| --- | --- | --- | --- | --- |
| Intercept (β₀) | 2.54 | 6.77 | 0.38 | 0.72 |
| tDCS Improvement (β₁) | 1.22 | 0.49 | 2.48 | 0.04 |
| Levodopa Improvement (β₂) | 1.26 | 0.37 | 3.04 | 0.02 |

**Supplementary table 1.** **Results of the linear model in which network tDCS and levodopa improvements were used as regressors to estimate DBS improvements.** The table summarizes estimated coefficients for the two variables which were both significant predictors.
